# Supplementary material for: Left Ventricular Diastolic Indices and Their Impact on Outcomes in Patients with Recently Diagnosed Atrial Fibrillation
Source: J Clin Med. 2022 Sep 28;11(19):5732. doi: 10.3390/jcm11195732 (PMC9571305; doi:10.3390/jcm11195732)
Supplement: Supplementary file 1 [file jcm-11-05732-s001.zip › jcm-1926623-Supplementary.pdf]

Supplemental Material

Supplemental Figure S1. The Distribution of the calculated LVDD scores.

Table S1. The Cox proportional hazards regression model for factors associated with MACNEs at 2 years after registration.

Table S2. Sensitivity analysis excluding patients with moderate or severe mitral valvular heart disease, left ventricular hypertrophy, and a cardiac pacemaker for a Cox regression analysis

Table S3. Baseline characteristics of patients with and without Atrial Fibrillation Effect on QualiTy of Life Data

Table S4. Factors Independently Associated With impaired HR-QoL at enrollment

Table S5. The Atrial Fibrillation Effects on QualiTy of Life outcomes across patients with and without LA dilatation.

Supplemental appendix. The list of the names who involved in the Keio interhospital Cardiovascular Studies – Atrial Fibrillation Registry

Supplemental Figure S1. The Distribution of the calculated LVDD scores.

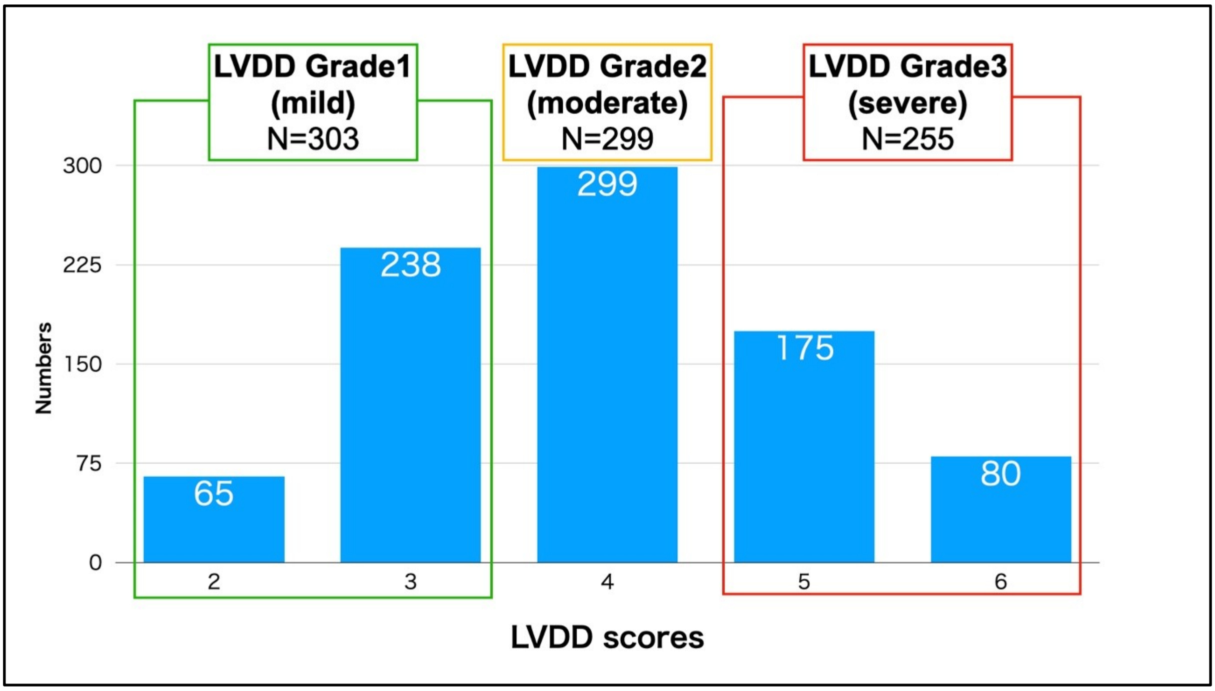

Abbreviations; LVDD, left ventricular diastolic dysfunction

Table S1. The Cox proportional hazards regression model for factors associated with MACNEs at 2 years after registration.

Factor	Hazard ratio	95% confidence interval		P Value
		Lower limit	Upper limit	
LVDD grade 1(vs grade 0)	1.10	0.44	2.76	0.82
LVDD grade 2(vs grade 0)	1.82	0.89	3.71	0.097
LVDD grade 3(vs grade 0)	2.28	1.13	4.60	0.021
Female (vs male)	1.15	0.67	1.96	0.60
Hypertension	1.31	0.71	2.40	0.37
Diabetes mellitus	1.73	0.96	3.10	0.065
Prior stroke or TIA	1.01	0.45	2.28	0.96
Prior CAD or PAD	1.09	0.52	2.27	0.81
Paroxysmal AF (vs others)	0.87	0.51	1.50	0.63
Use of OACs at registration	0.41	0.22	0.76	0.005
Age (per 1-year increase)	1.08	1.04	1.11	<0.001
BMI (per 1 increase)	0.91	0.83	0.99	0.037
eGFR (per 1 increase)	0.98	0.96	0.99	0.014
LA diameter (per 1cm increase)	1.43	0.99	2.07	0.054

Dependent variable; MACNE, major adverse cardiovascular or neurological events defined as a composite of all-cause death, stroke/non-CNS systemic embolism, and heart failure hospitalization.

The model stratified for the patients within sites, and was adjusted for clinically relevant factors as follows; LVDD grades (grade 0 as reference), sex, age (as contentious variables), hypertension, prior stroke or transient ischemic attack, coronary artery disease or peripheral artery disease, paroxysmal AF, use of oral anticoagulants at baseline, body mass index (as contentious variables), baseline estimated glomerular filtration rate (eGFR; as a continuous variable) and LA diameter (per 1cm increase).

Table S2. Sensitivity analysis excluding patients with moderate or severe mitral valvular heart disease, left ventricular hypertrophy, and a cardiac pacemaker for a Cox regression analysis

Factor	Hazard ratio	95% confidence interval		P Value
		Lower limit	Upper limit	
LVDD grade 1(vs grade 0)	1.27	0.55	2.90	0.58
LVDD grade 2(vs grade 0)	2.38	1.19	4.74	0.01
LVDD grade 3(vs grade 0)	1.81	0.85	3.89	0.13
Female (vs male)	0.86	0.49	1.49	0.58
Hypertension	1.27	0.71	2.29	0.42
Diabetes mellitus	0.91	0.49	1.71	0.77
Prior stroke or TIA	0.98	0.48	2.02	0.95
Prior CAD or PAD	0.80	0.36	1.78	0.58
Paroxysmal AF (vs others)	0.70	0.40	1.25	0.23
Use of OACs at registration	0.51	0.25	1.05	0.07
Age (per 1-year increase)	1.08	1.04	1.12	0.00
BMI (per 1 increase)	0.91	0.83	0.99	0.03
eGFR (per 1 increase)	0.98	0.97	1.00	0.02
LA diameter (per 1cm increase)	1.13	0.76	1.68	0.54

Dependent variables; MACNE, major adverse cardiovascular or neurological events defined as a composite of all-cause death, stroke/non-CNS systemic embolism, and heart failure hospitalization.

Abbreviations; LVDD, left ventricular diastolic dysfunction; TIA, transient ischemic attack; CAD, coronary artery disease; PAD, peripheral artery disease; OAC, oral anticoagulant; BMI, body mass index; eGFR, estimated glomerular filtration rate; LA, left atrium.

Table S3. Baseline characteristics of patients with and without Atrial Fibrillation Effect on Quality of Life Data

**Characteristics, no. (%)**	**Patients with AFEQT data n=1,520 (85.6%)**	**Patient without AFEQT data n=255 (14.4%)**	**P value**
Age, mean (SD), years	67.0 (10.8)	67.7 (13.9)	0.32
Men	1060 (69.7)	173 (67.8)	0.54
BMI, mean, kg/m^2 (SD)	23.7 (3.6)	23.0 (3.3)	0.004
Medical history			
Smoking	235 (15.5)	38 (15.0)	0.83
Hypertension	843 (55.5)	129 (50.6)	0.14
Diabetes mellitus	212 (13.9)	35 (13.7)	0.92
Dyslipidemia	536 (35.3)	64 (25.1)	0.001
Stroke or TIA	119 (7.8)	17 (6.7)	0.51
CKD (eGFR<60 ml/min)	581 (38.2)	81 (31.8)	0.048
Peripheral artery disease	46 (3.0)	6 (2.4)	0.55
Coronary artery disease	77 (5.1)	10 (3.9)	0.44
BNP, mean, pg/ml, (SD)	127.6 (139.7)	147.5 (169.1)	0.058
CHA_2DS_2-VASc score, mean (SD)	2.3 (1.6)	2.4 (1.7)	0.32
LVEF, mean, % (SD)	60.1 (3.7)	60.1 (4.1)	0.90
LA diameter, mean, cm (SD)	4.1 (0.7)	4.1 (0.8)	0.57
Type of visit			
Referral from emergency department	108 (7.1)	26 (10.2)	0.084
Diagnosed at health screening	435 (28.6)	78 (30.6)	0.52
Type of AF			
First detected	71 (4.7)	20 (7.9)	0.047
Paroxysmal	851 (56.0)	135 (53.1)	
Persistent	400 (26.3)	57 (22.4)	
Permanent	176 (11.6)	40 (15.7)	

Abbreviations; KiCS-AF, the Keio interhospital Cardiovascular Studies-atrial fibrillation; IQR, interquartile range; SD, standard deviation; BMI, body mass index; TIA, transient ischemic attack; CKD, chronic kidney disease; HD, hemodialysis; coronary artery bypass grafting; BNP, brain natriuretic peptide; LVEF, left ventricular ejection fraction; LA, left atrium.

Table S4. Factors Independently Associated With impaired HR-QoL at enrollment

Factor	Odds ratio	95% confidence interval		
		Lower limit	Upper limit	*P* Value
LVDD (any grade vs grade 0)	1.38	1.12	1.71	0.002
Female (vs male)	1.85	1.48	2.31	<0.001
Hypertension	0.86	0.69	1.07	0.192
Diabetes mellitus	0.72	0.53	0.96	0.027
Prior stroke or TIA	0.98	0.67	1.43	0.926
Prior CAD or PAD	1.48	1.02	2.15	0.036
Paroxysmal AF (vs others)	1.69	1.37	2.09	<0.001
Use of OACs at registration	1.49	1.14	1.95	0.003
Age (per 1-year increase)	0.98	0.97	0.99	0.022
BMI (per 1 increase)	1.00	0.97	1.03	0.772
eGFR (per 1 increase)	0.99	0.99	1.00	0.757
LA diameter (per 1cm increase)	0.95	0.82	1.10	0.539

Dependent variable; patients with impaired HR-QoL at enrollment (categorical variable); defined as AFEQT-OS score at baseline was less than 80.

Abbreviations; LVDD, left ventricular diastolic dysfunction; TIA, transient ischemic attack; CAD, coronary artery disease; PAD, peripheral artery disease; OAC, oral anticoagulant; BMI, body mass index; eGFR, estimated glomerular filtration rate; LA, left atrium.

Table S5. The Atrial Fibrillation Effects on QualiTy-of-Life outcomes across patients with and without LA dilatation.

Change within 1-year, mean (95% confidence interval)*	Patients with normal diastolic function (grade 0)	Patients with diastolic dysfunction (any grade)	*P* value
Patients with LA dilatation (LA diameter ≥40mm)			
Overall summary	7.3 (6.1-8.5)	7.4 (6.2-8.7)	0.87
Symptom	8.0 (6.7-9.3)	7.7 (6.3-9.1)	0.72
Daily activities	6.7 (5.2-8.1)	6.1 (4.5-7.6)	0.59
Treatment concerns	7.9 (6.6-9.1)	9.6 (8.3-10.9)	0.059
Treatment satisfaction	13.1 (11.1-15.0)	15.5 (13.4-17.5)	0.095
Patients without LA dilatation (LA diameter <40mm)			
Overall summary	12.6 (11.3-13.9)	9.9 (8.6-11.2)	0.005
Symptom	13.0 (11.4-14.6)	11.9 (10.3-13.4)	0.29
Daily activities	12.0 (10.4-13.7)	7.6 (6.0-9.2)	<0.001
Treatment concerns	13.0 (11.6-14.4)	11.8 (10.4-13.2)	0.23
Treatment satisfaction	16.4 (14.2-18.6)	13.5 (11.4-15.6)	0.060

* Changes in AFEQT score within 1 year were defined as AFEQT score at 1-year minus AFEQT score at baseline, and were compared between each group by using analysis of covariance adjusted for baseline AFEQT scores. A positive change represents improved QOL, and a negative change implies worsening QOL.

Supplemental appendix. The list of the names who involved in the KiCS-AF registry

Site investigators: Yukihiko Momiyama, Munehisa Sakamoto, Jun Fuse, Kojiro Tanimoto, Yoko Tanimoto, Yukinori Ikegami, Kohei Inagawa (National Hospital Organization Tokyo Medical Center). **Iwao Nakamura**, Jyunji Suzuki, Tomohiro Matsuhashi, Hiroshi Shiga (Hino Municipal Hospital). **Seiji Takatsuki**, Yoshiyasu Aizawa, Nobuhiro Nishiyama, Takahiko Nishiyama, Yoshinori Katsumata, Shin Kashimura, Akira Kunitomi, Kazuaki Nakajima, Taishi Fujisawa (Keio University School of Medicine). **Masahiro Suzuki**, Takaharu Katayama, Keisuke Matsumura, Tomohiko Ono, Hanako Tokuda, Ryutaro Yamaguchi, Hiroaki Tanaka (National Hospital Organization Saitama National Hospital). **Shigetaka Noma**, Takashi Yagi, Kenichiro Shimoji, Koji Ueno, Satoshi Mogi (Saiseikai Utsunomiya Hospital). **Takashi Koyama**, Shiro Ishikawa, Hideaki Kanki, Takashi Akima, Masahito Munakata, Kazutaka Miyamoto (Saitama City Hospital). **Hideo Mitamura**, Kazunori Moritani, Masaru Shibata, Toshimi Kageyama (Tachikawa Hospital). **Takahiro Oki**, Akiyasu Baba, Yoshinori Mano, Hiroaki Sukegawa (Tokyo Dental College Ichikawa General Hospital). **Kouji Negishi**, Takahiro Koura, Daisuke Shinmura, Kotaro Fukumoto, Hiroyuki Yamakawa (Yokohama Municipal Citizen's Hospital). **Keiichi Nagami**, Kazuhiro Oyamada, Kotaro Naitou, Keijiro Chiba (Keiyu Hospital). **Megumi Shimada** (Tokai University Oiso Hospital). **Makoto Akaishi** (Tokai University Tokyo Hospital)

Clinical coordinators: Aki Kato, Ikumi Koishi, Miho Matsuoka, Takako Nozaki, Hiroaki Nagayama, Chieko Tamura, Reiko Tamura, Junko Susa, Miho Umemura, and Itsuka Saito.
